# Effects of Hyperbaric Oxygen Therapy on Cerebral Activity in Stroke Patients Based on fNIRS

**DOI:** 10.3390/s26061794

**Published:** 2026-03-12

**Authors:** Haitao Zhang, Cien Zhou, Fangfang Sun

**Affiliations:** School of Automation, Hangzhou Dianzi University, Hangzhou 310018, China; 231060377@hdu.edu.cn (H.Z.); 232060131@hdu.edu.cn (C.Z.)

**Keywords:** stroke, hyperbaric oxygen therapy (HBOT), functional near-infrared spectroscopy (fNIRS), alternating hand-clenching paradigm, spatiotemporal dynamics

## Abstract

Stroke remains a leading cause of death and disability worldwide, imposing significant burdens on patients, families, and healthcare systems. Despite advances in acute management and rehabilitation, effective interventions to promote neural recovery remain limited. Hyperbaric oxygen therapy (HBOT) has emerged as a potential adjunctive treatment, but its effects on cortical functional activity—particularly the neurophysiological mechanisms underlying clinical improvements—remain insufficiently understood. This study aimed to investigate the effects of hyperbaric oxygen therapy (HBOT) on cerebral activation in stroke patients using functional near-infrared spectroscopy (fNIRS) and to evaluate its therapeutic efficacy. A total of 23 patients with intracerebral hemorrhage and 20 with cerebral infarction were enrolled. fNIRS data were collected before HBOT and within 10–30 min after treatment completion. During data acquisition, participants performed an alternating left- and right-hand grip task while wearing the fNIRS device throughout the procedure. Changes in near-infrared light intensity were monitored to objectively reflect cortical activity. The results showed that after HBOT, activation patterns in relevant brain regions during the grip task were significantly altered: activation channels during the bilateral grip task changed in cerebral infarction patients, with some brain regions overlapping with those observed in intracerebral hemorrhage patients. In intracerebral hemorrhage patients, the number of significantly activated channels decreased during the left-hand grip task but increased notably during the right-hand grip task, which may be related to cerebral functional compensation and right-hand dominance. Clinical assessments revealed significant post-treatment improvements in Brunnstrom stage, Fugl-Meyer scores, and activities of daily living. These findings suggest that HBOT may contribute to multifaceted recovery of brain function in stroke patients, not only by enhancing cerebral blood flow and oxygenation but also by facilitating neural repair and regeneration, as well as optimizing cerebral activation and functional connectivity. Thus, this study provides an objective basis for understanding the mechanisms and efficacy of HBOT in stroke rehabilitation.

## 1. Introduction

Stroke is a cerebrovascular disease characterized by high incidence, high disability rates, and high mortality rates, imposing a significant burden on patients themselves, their families, and the broader healthcare system [1]. The pathological basis lies in the interruption of cerebral blood flow, which leads to ischemic necrosis of neurons and dysfunction of associated neural networks, thereby resulting in various neurological deficits in motor, sensory, cognitive, and other functions [2]. Therefore, exploring effective interventions to promote the recovery of brain function and improve patients’ quality of life has become an important research focus in the field of neurological rehabilitation.

At the neuronal level, stroke initiates a series of pathological processes, including excitotoxicity, oxidative stress, inflammation, and apoptosis, leading to neuronal death in the core infarct region and functional disturbances in the surrounding penumbra and connected remote areas [3]. These alterations manifest as disruptions in neurovascular coupling—the physiological mechanism that links local neuronal activity to regional cerebral blood flow changes. In healthy brains, increased neuronal activity triggers a localized hemodynamic response, increasing oxygen delivery to active regions. After stroke, this coupling is often impaired due to endothelial dysfunction, pericyte damage, and altered metabolic demand, resulting in decoupling between neural activity and hemodynamic responses.

In recent years, hyperbaric oxygen therapy has garnered increasing attention as an adjunctive treatment in the clinical management of stroke. This therapy significantly enhances blood oxygen levels and diffusion distance in a hyperbaric oxygen environment, enabling ischemic brain tissues to receive more adequate oxygen supply [4,5]. Adequate oxygenation is believed to help improve the metabolic state of the injured area, alleviate hypoxic damage to neural cells, and thereby create favorable conditions for neural repair and regeneration [6,7]. However, despite the clinical application of hyperbaric oxygen therapy, its specific mechanisms of action on brain neural activity and its therapeutic efficacy in stroke patients still require further investigation.

Among neuroimaging techniques for assessing the effects of hyperbaric oxygen therapy, functional magnetic resonance imaging (fMRI) and positron emission tomography (PET) offer high spatial resolution but are limited by high equipment costs, sensitivity to motion artifacts, and challenges in real-time dynamic monitoring near the treatment chamber. While electroencephalography (EEG) is highly portable, its signals are susceptible to interference from electromyographic and ocular artifacts, and its spatial resolution is relatively limited [8]. In contrast, functional near-infrared spectroscopy (fNIRS), as a non-invasive method for brain functional imaging, indirectly reflects neural activity by detecting changes in hemoglobin concentrations within cortical blood-oxygen-level-dependent signals. It offers advantages such as strong resistance to motion artifacts, high temporal resolution, and portability, making it particularly suitable for continuous real-time monitoring of brain function in specialized environments such as hyperbaric oxygen therapy [9,10].

Based on the considerations above, this study employs fNIRS technology to investigate the effects of hyperbaric oxygen therapy on brain activity in stroke patients. In the study, participants performed an alternating hand-gripping task before and after hyperbaric oxygen therapy [11]. Subsequently, the spatiotemporal dynamics of oxyhemoglobin (HbO_2_) concentration in the motor cortex were analyzed to objectively evaluate the regulatory effects of hyperbaric oxygen therapy on cortical activation patterns. This research aims to elucidate the potential mechanisms of hyperbaric oxygen therapy in neurorehabilitation and provide an objective basis for optimizing clinical treatment strategies [12].

## 2. Materials and Methods

### 2.1. Ethics Statement

This study was approved by the Ethics Committee of Ningbo Rehabilitation Hospital (Approval No: Ningbo Rehabilitation Hospital, 2025, Ethical Review No. 2025-KY-015) in accordance with the Declaration of Helsinki. All participants provided written informed consent prior to enrollment after being fully informed of the study’s purpose, procedures, potential risks, and benefits.

### 2.2. Participants

All participants were recruited from Ningbo Rehabilitation Hospital. In this study, after applying the inclusion/exclusion criteria and conducting data quality screening, a total of 43 eligible participants were finally included. All enrolled patients were in the subacute to chronic recovery phase of stroke (2 weeks to 6 months post-onset) to ensure they could tolerate and cooperate with the experimental procedures. These 43 participants were further divided into two groups: 23 patients with intracerebral hemorrhage (17 males, 6 females; mean age 58.6 ± 8.0 years), and 20 patients with cerebral infarction (15 males, 5 females; mean age 60.5 ± 7.4 years). All patients received standard hyperbaric oxygen therapy; each HBOT session consisted of three phases: a compression phase (approximately 10–15 min) to reach a treatment pressure of 2.0 ATA, a maintenance phase (60 min) at 2.0 ATA during which patients breathed normally, and a decompression phase (approximately 10–15 min) to return to ambient pressure. The total session duration was approximately 80–90 min.

### 2.3. Experimental Paradigm Design

Prior to the start of the experiment, the participant was seated in a chair, and a brief overview of the experimental procedure was provided by the research assistant. Following this, a functional near-infrared spectroscopy (fNIRS) probe cap was fitted onto the participant’s head according to standard protocols, and the optodes were adjusted to minimize signal interference from hair, ensuring adequate scalp contact, thereby ensuring the completeness and stability of signal acquisition. Once the cap was properly positioned, resting-state data collection commenced.

The resting-state task began with an on-screen prompt stating “Please remain physically relaxed.” During this phase, participants were instructed to face the display and maintain fixation while minimizing additional cognitive and motor loads. The resting-state period lasted for 3 min.

Upon completion of the resting state, the screen displayed the message “Prepare for the upcoming task,” signaling the imminent start of the task phase. The preparation period was set to 10 s. After the preparation phase ended, simultaneous presentation of the text prompt “Perform continuous fist-clenching movements” and a demonstration video of fist clenching was provided. If participants exhibited delays in movement initiation or insufficient range of motion during the task, an assistant on-site would perform the standardized movement synchronously with the video to guide the participant in completing the task correctly. Each fist-clenching task lasted 10 s.

After the task concluded, the screen displayed the message “Maintain a resting state”, initiating a 10-s rest interval. Following the rest period, the prompt “Prepare for the upcoming task” reappeared, signaling the start of the next task cycle. This fist-clenching task was repeated for a total of 10 cycles [13].

**Figure 1:**
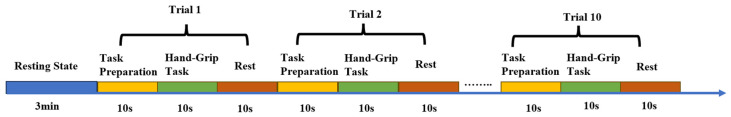
Experimental Paradigm Flowchart.

Throughout the experiment, an assistant recorded real-time markers based on the participant’s task performance to facilitate subsequent data segmentation and feature extraction. The experimental procedure is illustrated in Figure 1.

### 2.4. Experimental Signal Acquisition

Functional near-infrared spectroscopy (fNIRS) signals were acquired using the NirSmart-6000A fNIRS system (Danyang Huichuang Medical Technology Co., Ltd., Danyang City, China) with software version NirSpark 1.5, a continuous-wave, multi-channel system designed for clinical and research applications. The system employs three specific near-infrared wavelengths (730 nm, 808 nm, and 850 nm) to measure concentration changes of oxygenated hemoglobin (HbO_2_), deoxygenated hemoglobin (Hb), and total hemoglobin (tHb) based on the modified Beer-Lambert law.

**Figure 2:**
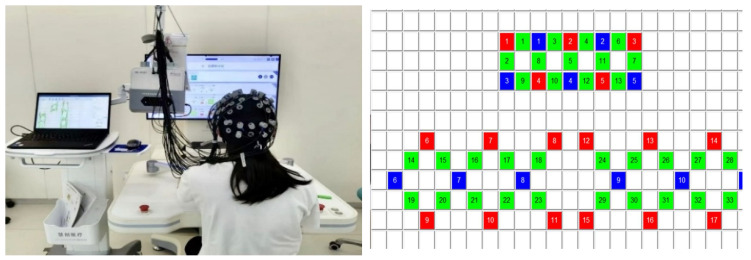
Experimental Environment and fNIRS Channel Layout.

The system utilizes a flexible probe cap with adjustable optode holders to accommodate different head sizes. A total of 33 data channels were used in this experiment, configured with a source-detector separation of 3.0 cm, which provides optimal sensitivity to cortical hemodynamic changes with minimal contamination from extracerebral tissues. The channel arrangement consisted of 13 channels covering the prefrontal cortex (Brodmann areas BA9, BA10, BA11, BA46) and 20 channels distributed over the bilateral motor cortex regions (Brodmann areas BA1-4, BA6), including the primary motor cortex, premotor cortex, and supplementary motor area. The specific channel locations and their corresponding MNI coordinates are provided in Figure 2. The red marks indicate the light source emitters, the blue marks indicate the detector receivers, and the green marks indicate the channels. Data were acquired at a sampling rate of 10 Hz, which is sufficient to capture hemodynamic responses while minimizing data storage requirements. Prior to each recording session, the optode positions were adjusted to ensure that hair beneath each channel was properly moved aside, and signal quality was verified by checking the gain levels and signal-to-noise ratio for each channel. Channels with poor signal quality (coefficient of variation > 15% during resting state) were excluded from analysis.

### 2.5. MNI Coordinates and Brodmann Areas

To precisely localize the cortical functional regions corresponding to the fNIRS channels, this study employed the internationally recognized standard brain space coordinate system—the Montreal Neurological Institute (MNI) standard space—for spatial normalization and performed functional anatomical interpretation with reference to the classic Brodmann area system.

#### 2.5.1. MNI Coordinate System

The MNI coordinate system is the most widely used standard brain coordinate system in current brain imaging research. Based on a standardized brain template, it locates any position within the brain via a three-dimensional Cartesian coordinate system (X, Y, Z axes). The X-axis represents the left-right direction (positive values indicate the right hemisphere, negative values the left hemisphere); the Y-axis represents the anterior-posterior direction (positive values indicate anterior, negative values posterior); and the Z-axis represents the superior-inferior direction (positive values indicate superior, negative values inferior). The unit of coordinates is millimeters (mm). This system effectively reduces the influence of individual differences in brain structure across subjects, enabling unified quantification and comparative analysis of brain regions. The MNI coordinates corresponding to the 33 fNIRS channels in the present study are listed in detail in Table 1.

#### 2.5.2. Brodmann Area System

Brodmann areas are a brain region parcellation scheme proposed by the German neurologist Korbinian Brodmann in 1909 [14]. Based on cytoarchitectonic differences in the cerebral cortex, this system divides the cortex into 52 distinct functional regions, each associated with specific brain functions such as sensation, movement, language, and cognition. Each region is labeled with a unique number (e.g., BA1, BA2), and different areas correspond to distinct functional localizations within the cerebral cortex.

In the present study, by matching the MNI coordinates of each fNIRS channel with the standard brain template, we determined the corresponding Brodmann areas and their proportions for each channel (the proportion indicates the contribution degree of the channel signal to a specific Brodmann area). The specific corresponding relationships are shown in Table 2.

### 2.6. Clinical Functional Assessment

Standardized clinical assessments were performed within 24 h before the first HBOT session and within 24 h after the final HBOT session. All assessments were conducted by the same two certified rehabilitation therapists, blinded to fNIRS results, to ensure consistency.

Three standardized measures were administered. Brunnstrom staging (upper limb): This ordinal scale (Stages I–VI) assesses motor recovery based on movement pattern evolution, from flaccid paralysis (Stage I) to near-normal isolated movements (Stage VI); Fugl-Meyer Assessment for upper extremity (FMA-UE): This 33-item scale evaluates upper limb motor function, coordination, and reflexes. Each item is scored 0–2 (maximum 66), with higher scores indicating better function; Barthel Index (ADL): This 10-item scale measures functional independence in self-care and mobility. Scores range from 0 (complete dependence) to 100 (complete independence).

All data were recorded on standardized forms. Assessors were not involved in HBOT administration and had no access to fNIRS data until study completion.

## 3. Data Processing

**Figure 3:**
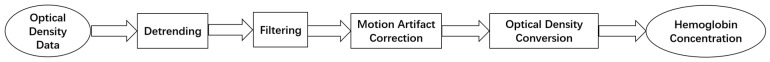
Preprocessing Workflow.

The preprocessing workflow adopted in this study is illustrated in Figure 3. During long-term fNIRS data acquisition, the signals are susceptible to various sources of interference, including variations in light-source coupling between the device probes and the scalp, subtle head movements of the participants, as well as physiological activities such as respiration and heartbeat. These artifacts are unrelated to task-evoked changes in hemoglobin concentration but become superimposed on the target signals, complicating the composition of cerebral hemodynamic responses. To effectively mitigate the impact of noise, systematic preprocessing of the raw signals is necessary. The steps specifically include detrending, filtering, motion artifact correction, and conversion of optical density data into hemoglobin concentration values based on the modified Lambert-Beer law [15]. The entire procedure was implemented using the NIRS-KIT toolbox within the MATLAB R2021a software environment [16].

## 4. Feature Extraction

The mean value of hemoglobin concentration is one of the most commonly used features in fNIRS studies, offering an intuitive reflection of brain activation states. It represents the average change in hemoglobin concentration over a time interval from t1 to t2. An increase or decrease in the mean value can provide a basic estimation of the overall activation level in brain regions of interest. The specific calculation method is shown in Equation (1).

(1) $$\mathrm{mean}=\frac{1}{t_{2}-t_{1}}\sum_{t_{1}}^{t_{2}}x_{t}$$

Existing research indicates that elevated levels of oxyhemoglobin (HbO_2_) are more directly associated with cerebral activation. Furthermore, within the near-infrared spectrum, the absorption of light at specific wavelengths by oxyhemoglobin exhibits relatively pronounced changes. Therefore, this study utilizes oxyhemoglobin concentration for subsequent analysis. Paired *t*-tests and Mann-Whitney U tests were employed to compare the mean oxyhemoglobin concentrations across channels for the same participant group (i.e., either the intracerebral hemorrhage group or the cerebral infarction group) under different task conditions (left-hand gripping vs. right-hand gripping). Multiple comparison correction was applied. Results with a *p*-value below 0.05 in either test were considered indicative of a significant difference in mean cerebral oxyhemoglobin levels between the two task conditions.

Statistical analysis was performed using paired *t*-tests and Mann-Whitney U tests to compare mean oxyhemoglobin concentrations across channels for the same participant group (intracerebral hemorrhage or cerebral infarction) under different task conditions (left-hand vs. right-hand gripping). Both parametric and non-parametric methods were employed to ensure robustness, as fNIRS data may deviate from normality assumptions. The *t*-test is more sensitive to normally distributed data, while the U-test is resistant to outliers and non-normal distributions. Multiple comparison correction was applied to control for Type I error. A *p*-value < 0.05 in either test was considered statistically significant, indicating a difference in cerebral activation between task conditions. Discrepancies between the two tests (presented in Table 3 and Table 4) reflect the influence of data distribution characteristics on statistical significance.

## 5. Results and Analysis

### 5.1. Results and Analysis Based on Mean Values

To further investigate the differences in cortical activation levels between patients with intracerebral hemorrhage and cerebral infarction under different task conditions, this study comparatively analyzed the changes in mean activation values across channels for both groups during left- and right-hand gripping tasks relative to the resting state. Table 3 and Table 4 respectively present the number of channels identified as showing significant changes and their corresponding anatomical locations before and after hyperbaric oxygen therapy, based on *t*-tests and U-tests.

### 5.2. Patients with Intracerebral Hemorrhage Performing Left-Hand Grip

Based on the MNI coordinates of each channel, this study visualized changes in mean activation using the EasyTopo toolbox running on MATLAB 2021a. In the resulting maps, red areas represent brain regions with increased average activation compared to the resting state, while blue areas indicate decreased average activation, with color intensity corresponding to the magnitude of change. To facilitate comparison of brain activation patterns across different hand-grip tasks, the statistical significance threshold for all tasks was uniformly set at 1 × 10^−7^.

**Figure 4:**
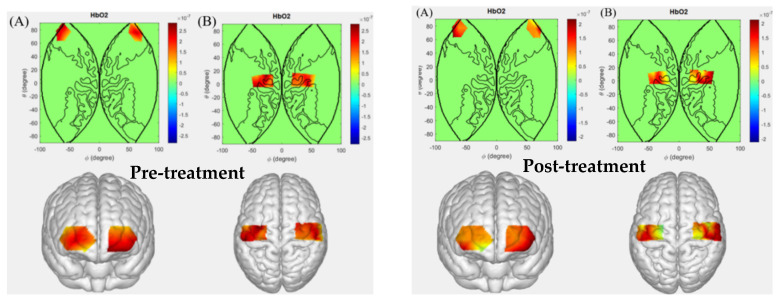
Cerebral activation levels in patients with intracerebral hemorrhage before and after hyperbaric oxygen therapy during the left-hand grip task. ((**A**): Prefrontal cortex; (**B**): Motor cortex).

During the left-hand gripping task, the cerebral activation levels in patients with intracerebral hemorrhage before and after hyperbaric oxygen therapy are shown in Figure 4. Comparative results indicate that after hyperbaric oxygen therapy, patients in the left-hand gripping task group exhibited altered activation patterns in channels Ch4, Ch6, Ch8, Ch9, and Ch31 compared to the pre-treatment state. These channels correspond to Brodmann areas BA1, BA2, BA3, BA4, BA6, BA9, BA10, and BA11.

Among these, BA1, BA2, and BA3 are associated with the somatosensory cortex. Intracerebral hemorrhage damages the existing sensorimotor circuits. Before treatment, likely due to lesion interference, the channels corresponding to these somatosensory cortical regions failed to participate normally in sensory information processing and motor coordination, resulting in no significant activation. After hyperbaric oxygen therapy, with improved cerebral blood circulation and optimized metabolic conditions for neural cells, neuronal functions in these regions gradually recovered, enabling them to once again receive and process sensory information from the left-hand gripping task and engage in effective information exchange with motor-related brain areas.

BA4 and BA6 correspond to the motor cortex, involving motor execution and motor planning functions, respectively. Following intracerebral hemorrhage, the engagement of these regions in motor planning and preparation for the left-hand gripping task was impaired, manifesting as significantly reduced activation in the associated channels. After hyperbaric oxygen therapy, as brain function gradually recovered, these areas were able to participate more effectively in the planning and preparation processes of the left-hand gripping action. By integrating sensory feedback from the hand and motor goals, they adjusted motor commands and coordinated with other motor-related brain regions, leading to noticeable activation changes in the corresponding channels, which indicates restored functionality in the control process of the left-hand gripping action.

BA9, BA10, and BA11 are associated with higher cognitive and executive functions. Intracerebral hemorrhage may disrupt overall brain functional coordination, limiting the supportive role of these higher cognitive regions in the left-hand gripping task. After hyperbaric oxygen therapy, with overall brain function improvement and restored neural network information transmission and integration capabilities, these regions regained their involvement in the left-hand gripping task. For instance, they enhanced attentional modulation of the action and refined movement execution based on motor memory, resulting in activation changes in the corresponding channels, which reflect their restored participation and regulatory functions in the left-hand gripping task.

### 5.3. Patients with Intracerebral Hemorrhage Performing Right-Hand Grip

**Figure 5:**
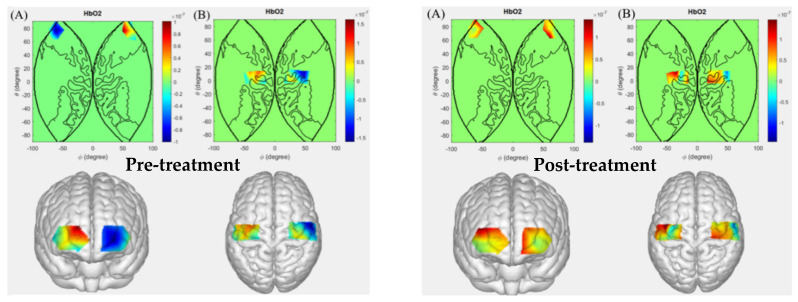
Cerebral activation levels in patients with intracerebral hemorrhage before and after hyperbaric oxygen therapy during the right-hand grip task ((**A**): Prefrontal cortex; (**B**): Motor cortex).

Figure 5 presents a comparative analysis of cerebral activation levels in patients with intracerebral hemorrhage before and after hyperbaric oxygen therapy during the right-hand gripping task. Following treatment, patients exhibited altered activation patterns in channels Ch3, Ch5, Ch12, Ch13, Ch19, Ch20, Ch23, Ch29, Ch30, and Ch32 compared to pre-treatment. These channels correspond to Brodmann areas BA1, BA2, BA3, BA4, BA6, BA10, BA11, and BA40.

The findings indicated that BA1, BA2, and BA3 are involved in somatosensory cortical functions. Intracerebral hemorrhage can disrupt normal neural pathways and neuronal function. Prior to treatment, factors such as local hemorrhagic injury and cerebral edema may have prevented these somatosensory regions from effectively receiving and processing sensory information generated during right-hand gripping, resulting in no significant activation in the corresponding channels. After hyperbaric oxygen intervention, with improved cerebral blood flow perfusion, reduced cerebral edema, and optimized neuronal metabolism, neuronal function in these regions gradually recovered. This enabled accurate perception of proprioceptive and tactile information from the right hand, such as proprioceptive feedback from muscle contraction and relaxation as well as tactile signals from external contact. The restored sensory information could then be transmitted to motor-related brain regions, contributing to the fine-tuning of the right-hand gripping action.

BA4 and BA6 correspond to motor cortical regions. Following intracerebral hemorrhage, overall motor planning and coordination functions were impaired, leading to a lack of significant activation in BA4 and BA6 during the right-hand gripping task, indicating reduced functional engagement in motor execution. After hyperbaric oxygen therapy, as brain function gradually recovered, these regions showed increased involvement in the planning and preparation phases of the right-hand gripping action. They were able to adaptively adjust motor output commands based on proprioceptive feedback and anticipated movement goals while enhancing coordination with other motor-related brain areas. Correspondingly, significant activation changes were observed in the related channels, demonstrating the restoration of regulatory functions of BA4 and BA6 in the control process of the right-hand gripping movement.

BA10 and BA11 are key brain regions involved in higher cognitive and executive functions. After intracerebral hemorrhage, impaired overall brain functional coordination may have hindered their effective auxiliary regulatory role in the right-hand gripping task. Following hyperbaric oxygen therapy, with overall improvement in brain function, particularly the restoration of interregional information transmission and integration, these cognitive-related regions regained participation in the regulation of the right-hand gripping task. Specifically, patients demonstrated enhanced attention during task execution and optimized grip strength and rhythm based on prior experience. The observed activation changes in the corresponding channels further confirm the involvement of these regions in the task and their contribution to movement optimization post-recovery.

The BA40 region is involved in critical functions such as sensory integration and spatial cognitive processing. Intracerebral hemorrhage may impair its ability to integrate sensory information and process spatial cognition, resulting in no significant activation in the corresponding neural channels before treatment. After hyperbaric oxygen intervention, as brain function gradually recovered, the BA40 region regained its capacity to effectively integrate multiple sensory inputs during right-hand gripping and accurately identify the hand’s spatial position and movement state, thereby enhancing the precision of motor execution. The observed activation changes in the related channels indicate the restoration and involvement of this region in the sensory-spatial coordination function of the right-hand gripping action.

### 5.4. Patients with Cerebral Infarction Performing Left-Hand Grip

**Figure 6:**
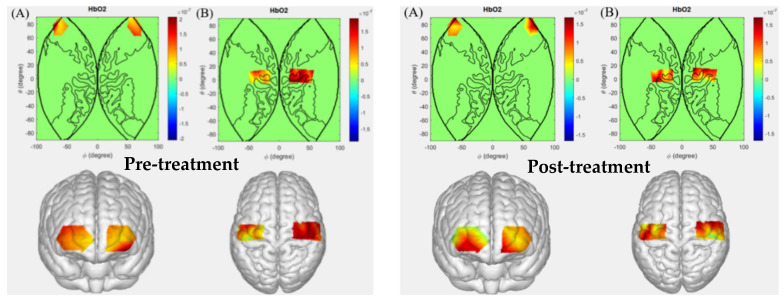
Cerebral activation levels in patients with cerebral infarction before and after hyperbaric oxygen therapy during the left-hand grip task ((**A**): Prefrontal cortex; (**B**): Motor cortex).

Figure 6 illustrates the comparative results of cerebral activation levels in patients with cerebral infarction before and after hyperbaric oxygen therapy during the left-hand gripping task. Following treatment, altered activation patterns were observed in channels Ch2, Ch6, Ch11, Ch18, Ch19, Ch20, and Ch23 compared to pre-treatment. These channels correspond to Brodmann areas BA1, BA2, BA3, BA4, BA6, BA10, BA11, BA46, and BA47. Among these, BA1, BA2, and BA3 are associated with the somatosensory cortex, and the findings further support the functional role of these regions in sensorimotor processing. Cerebral infarction leads to localized disruption of cerebral blood flow, resulting in ischemic and hypoxic damage, which impairs the ability of somatosensory cortical regions to receive and process sensory information. Prior to treatment, even during left-hand gripping, the conduction and integration efficiency of sensory information were reduced due to compromised neuronal function, and no significant activation was observed in the corresponding neural pathways. After hyperbaric oxygen intervention, with improved cerebral perfusion and optimized metabolic conditions for damaged neurons, the functionality of these cortical regions gradually recovered. They were able to accurately perceive sensory inputs from the left hand and relay this information to motor-related brain areas, thereby contributing to the fine-tuning of the left-hand gripping action.

BA4 and BA6 correspond to key components of the motor cortex. Following cerebral infarction, some neurons in these regions may undergo necrosis or functional impairment due to ischemia, and neural connections with other brain areas may also be disrupted. Consequently, prior to treatment, signal activation in the corresponding channels of these regions was significantly suppressed during the left-hand gripping task, making it difficult to properly regulate hand muscle movements. After hyperbaric oxygen intervention, surviving neurons may gradually restore their control over the target muscles through compensatory mechanisms. Simultaneously, improvements in cerebral blood flow and oxygen supply facilitate the repair of damaged neural network connections, enabling BA4 and BA6 regions to regain effective control over the left-hand gripping action. Correspondingly, significant changes in activation were observed in the associated signal channels, indicating the restoration of their core role in motor control during the left-hand gripping task.

BA10 and BA11 are critical brain regions involved in higher cognitive and executive functions. Following cerebral infarction, their ability to participate in overall functional coordination may be compromised, limiting their auxiliary regulatory role in the left-hand gripping task. After hyperbaric oxygen therapy, with overall improvement in brain function, particularly the restoration of information transmission and integration between brain regions, these higher cognitive-related regions regained their involvement in regulating the left-hand gripping task. Specifically, patients demonstrated more focused attention allocation during task execution and optimized parameters such as grip strength and rhythm based on motor experience, leading to significant activation changes in the corresponding neural channels. This phenomenon reflects the re-engagement of BA10 and BA11 in the left-hand gripping task and their role in optimizing motor execution post-recovery.

The BA46 and BA47 regions are involved in functions such as higher cognition, working memory, and social cognition. Cerebral infarction impairs the functional connectivity of related brain networks, preventing BA46 and BA47 from effectively participating in the coordinated regulation of the left-hand gripping task. After hyperbaric oxygen intervention, overall brain function improved in patients, and the capacity for neural network information transmission was restored. These higher cognitive-related brain regions re-established functional connections with the motor cortex and integrated their cognitive regulatory functions into the left-hand gripping action—for instance, facilitating the recall and adjustment of parameters such as grip strength and movement patterns. The observed neural activation changes in the corresponding pathways confirm the potential regulatory role of these brain regions in the left-hand gripping task following functional recovery.

### 5.5. Patients with Cerebral Infarction Performing Right-Hand Grip

**Figure 7:**
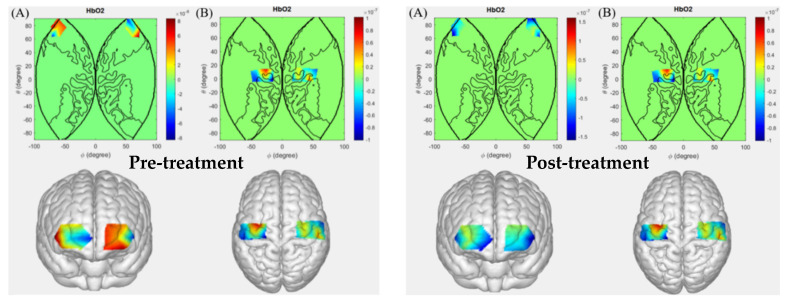
Cerebral activation levels in patients with cerebral infarction before and after hyperbaric oxygen therapy during the right-hand grip task. ((**A**): Prefrontal cortex; (**B**): Motor cortex).

Figure 7 illustrates the cerebral activation levels in patients with cerebral infarction before and after hyperbaric oxygen therapy during the right-hand grip task. Comparative analysis reveals that following hyperbaric oxygen therapy, patients with cerebral infarction exhibited altered activation patterns in channels Ch1, Ch2, Ch5, Ch7, Ch12, Ch14, Ch16, Ch17, Ch20, and Ch23 during the right-hand grip task, compared to pre-treatment conditions. These channels correspond to Brodmann areas BA1, BA2, BA3, BA4, BA6, BA10, BA11, BA46, and BA47. Notably, this activation distribution aligns with the brain regions involved in the left-hand grip task among cerebral infarction patients. Post-treatment, patients demonstrated gradual improvements in limb motor function, enhanced strength in the paretic limb, and increased fine motor skills of the hand. These observations suggest that previously impaired or functionally suppressed brain regions progressively regained normal levels of activation.

### 5.6. Mechanistic Comparison of Hyperbaric Oxygen Therapy Response Between Intracerebral Hemorrhage and Cerebral Infarction Patients

Although hyperbaric oxygen therapy can improve motor cortex activation in both intracerebral hemorrhage and cerebral infarction patients, significant differences exist in their underlying neural mechanisms: (1) Nature of Injury: Intracerebral hemorrhage primarily involves reversible damage dominated by mechanical compression, while cerebral infarction is characterized by irreversible ischemic necrosis. Consequently, post-treatment activation recovery in hemorrhage patients often concentrates in the primary injury area (e.g., the primary motor cortex BA4 or premotor area BA6), whereas infarction patients rely more on functional compensation from surrounding brain regions (e.g., adjacent areas such as BA46 or BA47 participating in motor regulation). (2) Oxygen Metabolic Demand: Due to insufficient collateral circulation, cerebral infarction patients exhibit greater dependence on hyperbaric oxygen to enhance local oxygen supply. This is reflected in a more pronounced increase in post-treatment motor cortex activation intensity (e.g., a larger rise in mean oxyhemoglobin concentration), indicating heightened sensitivity of ischemic brain tissue to improved oxygenation. (3) Functional Network Reorganization Patterns: In hemorrhage patients, post-treatment activation is primarily distributed within the sensorimotor network (BA1–BA6). In contrast, infarction patients additionally activate brain regions involved in cognitive-motor integration (e.g., BA46, BA47), which may be related to the more complex demands for large-scale neural network reorganization following infarction. These differences suggest that hyperbaric oxygen therapy protocols for stroke should be tailored based on etiology: cerebral infarction patients may benefit from extended treatment durations to promote collateral circulation formation, while hemorrhage patients are more suited to early intervention to alleviate edema compression and accelerate neurological recovery.

### 5.7. Correlation Analysis Between Brain Lesion Location and Functional Activation Patterns

To further investigate the impact of brain lesion location on therapeutic response, patients were categorized into three groups based on clinical data. The changes in cerebral activation patterns before and after treatment for these groups are presented in Table 5.

To further investigate the impact of brain lesion location on the response to hyperbaric oxygen therapy, this study categorized participants into three groups based on neuroimaging data and clinical presentations: the motor cortex pathway impairment group, the somatosensory cortex pathway impairment group, and the prefrontal-subcortical impairment group. By comparing changes in the number and spatial distribution of fNIRS-activated channels before and after hyperbaric oxygen therapy across these groups, this study observed that the therapy-induced remodeling of brain activity exhibited significant pathway specificity. In the motor cortex pathway impairment group, significant compensatory activation (*p* < 0.01) was observed in the ipsilateral motor cortex and ipsilateral cerebellum after treatment, with an average increase of 5.3 activated channels. This suggests that motor function recovery may primarily rely on compensatory mechanisms within ipsilateral neural pathways. In the somatosensory cortex pathway impairment group, post-treatment activation in the contralateral motor cortex showed spatial convergence, while compensatory activation emerged in sensory association areas (e.g., BA5, BA7) (*p* < 0.05), with an average increase of 4.1 activated channels. This indicates an enhancement in sensory-motor integration following hyperbaric oxygen therapy. In the prefrontal-subcortical impairment group, the activation range of the prefrontal cortex significantly expanded after treatment, accompanied by strengthened functional connectivity with the motor cortex (*p* < 0.01) and an average increase of 6.7 activated channels. This reflects an improvement in higher-order cognitive control functions.

The brain functional remodeling induced by hyperbaric oxygen therapy demonstrates notable neural pathway-dependent differences. Specifically, in patients with motor pathway impairments, the therapy primarily elicited compensatory activation in ipsilateral brain regions; in those with sensory pathway impairments, it enhanced sensory-motor integration and promoted compensatory involvement of sensory association cortices [17]. And in patients with prefrontal-subcortical pathway involvement, it improved brain functions related to higher-order cognitive control. These findings indicate that hyperbaric oxygen therapy does not elicit generalized enhancement of whole-brain activity; instead, it selectively promotes functional reorganization within specific neural networks associated with the impaired pathways [18].

### 5.8. Clinical Functional Assessment and Integrated Analysis

To evaluate the clinical efficacy of hyperbaric oxygen therapy in promoting neurological recovery in patients, this study implemented standardized clinical functional assessments before and after each fNIRS data collection session. All assessments were conducted by professional rehabilitation therapists on patients who were awake and cooperative. Three standardized functional measures were administered: the Brunnstrom staging for the upper limb, the Fugl-Meyer Assessment for upper extremity motor function (FMA-UE), and the Activities of Daily Living (ADL) scale. Paired-sample *t*-tests were used to compare clinical scores before and after hyperbaric oxygen therapy (with the Wilcoxon signed-rank test applied to Brunnstrom staging data). Detailed results are presented in Table 6.

As shown in Table 6, patients demonstrated statistically significant improvements in all three clinical functional assessments after treatment compared to pre-treatment (*p* < 0.001). Specifically, the Brunnstrom stage increased by an average of 0.6 stages, indicating a transition in motor patterns from associated reactions and synergistic movements toward isolated movements, reflecting optimized motor control; the Fugl-Meyer upper limb score improved by an average of 6.9 points, suggesting substantial progress in fine motor functions such as joint mobility, coordination, and reflex modulation of the upper limb; and the ADL score increased by an average of 12.7 points, confirming clinically meaningful enhancements in daily self-care abilities, including dressing, eating, and toileting.

The observed synchronous improvements across multiple clinical functions following hyperbaric oxygen therapy in this study are consistent with the changes in brain neural activity monitored via fNIRS. Enhancements in Brunnstrom staging and Fugl-Meyer scores signify the recovery of motor neural network function, which is directly reflected in neuroimaging as increased activation and normalized activation patterns in the primary motor cortex (BA4) and premotor area (BA6). For instance, the increase in the number of fNIRS-activated channels in patients with intracerebral hemorrhage during right-hand gripping tasks provides direct neural evidence supporting their elevated Fugl-Meyer scores. Improvements in activities of daily living are closely associated with activation of the prefrontal cortex (including regions such as BA9, BA10, and BA11). The functional recovery of these higher cognitive areas—involved in motor planning, initiation, and sustained attention—serves as a critical foundation for performing complex functional tasks. In summary, the findings of this study indicate that hyperbaric oxygen therapy not only promotes local functional reorganization in sensorimotor cortices but also enhances the coordinated involvement of higher cognitive regions such as the prefrontal cortex during motor tasks. This dual effect drives comprehensive recovery in stroke patients at both neurophysiological and clinical functional levels.

## 6. Discussions

### 6.1. Mechanistic Discussion

As noted by Efrati and Ben-Jacob [19], hyperbaric oxygen therapy may induce neuroplasticity through multiple mechanisms including improved mitochondrial function and reduced neuroinflammation. The brain functional remodeling observed following HBOT in our study demonstrates notable neural pathway-dependent differences. Specifically, in patients with motor pathway impairments, the therapy primarily elicited compensatory activation in ipsilateral brain regions; in those with sensory pathway impairments, it enhanced sensory-motor integration and promoted compensatory involvement of sensory association cortices. In patients with prefrontal-subcortical pathway involvement, it improved brain functions related to higher-order cognitive control.

Furthermore, Grefkes and Ward [20] emphasized that post-stroke cortical reorganization involves complex patterns of both local and distant network changes, which aligns with our observation of pathway-specific remodeling following HBOT. In our study, patients with different lesion locations exhibited distinct patterns of activation recovery: motor pathway impairments showed ipsilateral compensatory activation, sensory pathway impairments demonstrated enhanced sensory-motor integration, and prefrontal-subcortical injuries exhibited expanded prefrontal activation. These findings are consistent with the notion that post-stroke recovery involves reorganization of distributed neural networks rather than isolated regional changes.

The differential responses between intracerebral hemorrhage and cerebral infarction patients observed in our study provide insights into the mechanisms of HBOT. Hemorrhage patients, whose injury is primarily characterized by mechanical compression, showed post-treatment activation concentrated in the primary injury area (BA4, BA6), suggesting resolution of edema and restoration of function in compromised but viable tissue. In contrast, infarction patients, who suffer from irreversible ischemic necrosis, relied more on functional compensation from surrounding regions (BA46, BA47) and demonstrated greater dependence on improved oxygen supply, as reflected in more pronounced increases in motor cortex activation intensity.

These findings indicate that hyperbaric oxygen therapy does not induce broad, nonspecific enhancement of whole-brain activity but rather selectively facilitates functional reorganization within specific neural networks associated with the impaired pathways. The observed pathway specificity suggests that HBOT may work synergistically with endogenous neuroplasticity mechanisms to promote targeted recovery based on the site of injury. This aligns with the concept that rehabilitation interventions should be tailored to the specific neural deficits of individual patients.

Several potential mechanisms may underlie the observed HBOT effects. First, improved oxygen delivery may enhance mitochondrial function and energy metabolism in compromised neurons, supporting synaptic plasticity and neuronal survival. Second, reduced hypoxia may decrease neuroinflammation and oxidative stress, creating a more favorable environment for neural repair. Third, HBOT may promote angiogenesis and collateral circulation development, particularly in ischemic tissue, improving long-term oxygen and nutrient supply [21]. Fourth, the therapy may modulate neurotrophic factors and signaling pathways involved in synaptic plasticity and axonal sprouting. Future studies combining neuroimaging with molecular and cellular analyses could further elucidate these mechanisms [22].

### 6.2. Clinical Implications

The findings of this study have several important clinical implications. First, the correlation between improved cortical activation and enhanced functional outcomes provides objective evidence supporting the use of HBOT as an adjunctive rehabilitation strategy for stroke patients. The observed improvements in Brunnstrom stages (mean increase of 0.6 stages), Fugl-Meyer scores (mean increase of 6.9 points), and ADL scores (mean increase of 12.7 points) represent clinically meaningful changes that are likely to enhance patients’ quality of life.

Second, the pathway-specific response patterns suggest that HBOT protocols should be personalized based on stroke etiology and lesion location. Cerebral infarction patients, who exhibit greater dependence on improved oxygen supply and compensatory mechanisms, may benefit from extended treatment durations to promote collateral circulation development and network reconstruction. In contrast, intracerebral hemorrhage patients may require early intervention to alleviate edema compression and facilitate functional recovery in key brain regions. This etiologically tailored approach could optimize treatment outcomes and resource utilization.

Third, the involvement of higher cognitive regions in motor recovery following HBOT highlights the importance of considering cognitive function in motor rehabilitation. The observed correlation between prefrontal activation and ADL improvements suggests that interventions targeting cognitive-motor integration may enhance functional outcomes. Combining HBOT with cognitive rehabilitation or motor imagery training could potentially synergize with the therapy’s effects on prefrontal function.

Fourth, fNIRS provides quantitative, objective measurements of cortical activation that can serve as neurophysiological biomarkers for treatment response [23]. Unlike subjective clinical scales, which may be influenced by patient motivation or therapist variability, fNIRS signals offer an unbiased assessment of brain function. In this study, the observed correlation between increased motor cortex activation and improved Fugl-Meyer scores demonstrates that fNIRS can objectively validate functional improvements reported by patients and clinicians. Furthermore, the portability and ease of use of fNIRS make it suitable for repeated measurements throughout the rehabilitation process. Unlike fMRI, which requires patients to be transported to specialized facilities, fNIRS can be deployed at the bedside or in rehabilitation gyms, enabling frequent monitoring of cortical changes. This allows clinicians to track recovery trajectories in real time and adjust treatment protocols dynamically based on neurophysiological responses [24].

### 6.3. Limitations

This study has several limitations that should be considered when interpreting the findings.

First, the absence of a control group limits the causal attribution of the observed changes to HBOT, as spontaneous recovery and time-dependent plasticity may also contribute to the improvements. Patients in the subacute to chronic phase of stroke (2 weeks to 6 months post-onset) naturally experience some degree of functional recovery due to endogenous neuroplasticity. Without a comparison group receiving standard rehabilitation without HBOT, we cannot definitively isolate the therapy-specific effects. We have therefore moderated our causal language throughout the manuscript, using terms such as “associated with” rather than “promotes” to reflect this limitation.

Second, the relatively small sample size (n = 43) from a single center limits the generalizability of our findings. The observed pathway-specific patterns require validation in larger, multicenter studies with more diverse patient populations. Such studies could also enable subgroup analyses based on factors such as age, gender, lesion size, and time post-stroke, which may influence treatment response.

Third, the inclusion window of 2 weeks to 6 months post-stroke introduces variability in the neurological recovery phase. Patients in the subacute phase (2 weeks to 3 months) may experience more rapid spontaneous recovery and greater neural plasticity, whereas those in the chronic phase (3–6 months) may rely more on compensatory mechanisms. This temporal heterogeneity could influence both baseline cortical activation patterns and the magnitude of HBOT-associated changes. Future studies should consider stratifying patients by time post-stroke to better understand these differential effects and to identify optimal treatment windows.

Fourth, the lack of long-term follow-up prevents assessment of whether the observed improvements are sustained over time. Future studies should include follow-up assessments at multiple time points (e.g., 3, 6, and 12 months post-treatment) to evaluate the durability of HBOT effects.

Despite these limitations, our findings provide preliminary evidence for the association between HBOT and improved cortical function in stroke patients and lay the groundwork for future randomized controlled trials [25].

## 7. Conclusions

This study demonstrates that hyperbaric oxygen therapy is associated with significant changes in cortical activation patterns during motor tasks in stroke patients, as measured by fNIRS. These changes involve not only primary sensorimotor regions but also higher cognitive areas, and correlate with improvements in motor function and activities of daily living. The observed neural remodeling exhibits pathway specificity depending on lesion location, suggesting that HBOT may promote targeted rather than generalized recovery. These findings offer objective neurophysiological evidence supporting the clinical efficacy of HBOT in stroke rehabilitation. Future research should focus on personalized treatment protocols based on stroke etiology and lesion location and investigate the combination of HBOT with other rehabilitation modalities to optimize functional outcomes.

## Figures and Tables

**Table 1:** MNI Coordinates of Each Channel.

Channel	X (mm)	Y (mm)	Z (mm)	Channel	X (mm)	Y (mm)	Z (mm)
Ch1	−36.909	61.405	−15.8	Ch18	−19.98	−4.271	77.095
Ch2	−49.314	52.632	−0.63518	Ch19	−59.312	−27.448	53.239
Ch3	−10.808	69.15	−16.099	Ch20	−52.518	−26.987	63.059
Ch4	15.535	68.772	−15.143	Ch21	−43.073	−26.507	68.706
Ch5	2.7718	70.555	−1.8321	Ch22	−32.398	−25.435	73.347
Ch6	40.094	60.664	−14.239	Ch23	−20.701	−26.071	77.376
Ch7	49.805	51.245	−0.16077	Ch24	22.861	−0.59311	74.834
Ch8	−27.606	68.604	−1.8545	Ch25	35.358	−1.364	65.886
Ch9	−38.615	59.586	16.23	Ch26	44.834	−1.0439	60.313
Ch10	−13.138	71.129	16.147	Ch27	56.272	−1.8706	51.544
Ch11	30.461	66.68	−2.1316	Ch28	62.484	−2.709	39.983
Ch12	16.841	69.921	16.074	Ch29	22.551	−24.71	76.87
Ch13	41.077	58.47	14.91	Ch30	34.414	−23.983	72.973
Ch14	−59.254	−6.0835	47.576	Ch31	44.927	−23.861	67.31
Ch15	−50.97	−3.9333	56.165	Ch32	55.443	−24.5	58.323
Ch16	−41.861	−3.5054	63.365	Ch33	63.917	−27.722	48.956
Ch17	−31.739	−4.1798	68.142	-	-	-	-

**Table 2:** Proportions of Brodmann Areas Corresponding to Each Channel.

Channel	BAS	Proportion	Channel	BAS	Proportion	Channel	BAS	Proportion
1	BA10	0.0478	14	BA1	0.0076	25	BA8	0.0072
1	BA11	0.4019	14	BA3	0.178	26	BA4	0.1004
1	BA46	0.0957	14	BA4	0.3485	26	BA6	0.8996
1	BA47	0.4545	14	BA6	0.4432	27	BA4	0.1089
2	BA10	0.0161	14	BA43	0.0227	27	BA6	0.8911
2	BA45	0.0321	15	BA4	0.1673	28	BA1	0.0106
2	BA46	0.9518	15	BA6	0.8327	28	BA3	0.1095
3	BA10	0.0037	16	BA4	0.1202	28	BA4	0.2615
3	BA11	0.9963	16	BA6	0.8798	28	BA6	0.3887
4	BA11	1	17	BA6	1	28	BA43	0.2297
5	BA10	0.8413	18	BA6	1	29	BA4	0.6505
5	BA11	0.1587	19	BA1	0.2896	29	BA6	0.3495
6	BA10	0.0958	19	BA2	0.112	30	BA3	0.0099
6	BA11	0.2833	19	BA3	0.3514	30	BA4	0.6601
6	BA46	0.1417	19	BA40	0.2471	30	BA6	0.33
6	BA47	0.4792	20	BA1	0.3811	31	BA1	0.0149
7	BA45	0.0435	20	BA2	0.041	31	BA3	0.3941
7	BA46	0.9565	20	BA3	0.5287	31	BA4	0.5725
8	BA10	0.3716	20	BA4	0.0492	31	BA6	0.0186
8	BA11	0.6284	21	BA1	0.0655	32	BA1	0.4402
9	BA10	0.3279	21	BA3	0.3964	32	BA2	0.0039
9	BA46	0.6721	21	BA4	0.5382	32	BA3	0.4788
10	BA10	1	22	BA3	0.0167	32	BA4	0.12
11	BA10	0.3345	22	BA4	0.7525	32	BA40	0.0201
11	BA11	0.6622	22	BA6	0.2308	33	BA1	0.4389
11	BA47	0.034	23	BA4	0.7717	33	BA2	0.0789
12	BA10	1	23	BA6	0.2283	33	BA3	0.0824
13	BA10	0.3658	24	BA6	1	33	BA40	0.3548
13	BA46	0.6342	25	BA6	0.9928			

**Table 3:** Channels Exhibiting Significant Mean Value Changes in Hand-Grip Tasks Before and After Hyperbaric Oxygen Therapy Based on *t*-Test.

Number of Channels with Significant Changes	Before Hyperbaric Oxygen Therapy	After Hyperbaric Oxygen Therapy
*t*-Test for the Intracerebral Hemorrhage Group—Left-Hand Grip Task	A total of 17 channels (1,2,3,7,8,11,12,18,20,21,23,26,27,28,29)	A total of 7 channels (2,6,9,12,28,31,33)
*t*-Test for the Intracerebral Hemorrhage Group—Right-Hand Grip Task	A total of 4 channels (16,17,21,26)	A total of 5 channels (12,13,15,23,29)
*t*-Test for the Cerebral Infarction Group—Left-Hand Grip Task	A total of 12 channels (1,5,7,13,16,24,26,27,28,29,32,33)	A total of 8 channels (2,6,11,19,20,24,26,27)
*t*-Test for the Cerebral Infarction Group—Right-Hand Grip Task	A total of 0 channels	A total of 6 channels (2,7,12,14,20,23)

**Table 4:** Channels Exhibiting Significant Mean Value Changes in Hand-Grip Tasks Before and After Hyperbaric Oxygen Therapy Based on the U-Test.

Number of Channels with Significant Changes	Before Hyperbaric Oxygen Therapy	After Hyperbaric Oxygen Therapy
U-Test for the Intracerebral Hemorrhage Group—Left-Hand Grip Task	A total of 30 channels (1,2,3,5,6,7,8,9,11,12,13,14,16,17,18,19, 20,21,22,23,24,25,26,27,28,29,30,32,33)	A total of 19 channels (1,2,4,6,7,9,10,11,14,20,21, 24,25,26,27,28,29,31,33)
U-Test for the Intracerebral Hemorrhage Group—Right-Hand Grip Task	A total of 6 channels (7,11,15,16,17,21)	A total of 12 channels (3,5,12,13,15,16,19,20,23,29,30,32)
U-Test for the Cerebral Infarction Group—Left-Hand Grip Task	A total of 16 channels (1,4,5,7,10,12,13,15,16,24,26,27,28,29,32,33)	A total of 15 channels (1,2,4,6,11,16,18,19,20, 23,24,26,27,28,33)
U-Test for the Cerebral Infarction Group—Right-Hand Grip Task	A total of 4 channels (7,29,31,32)	A total of 12 channels (1,2,5,7,12,14,16,17,19,20,23,29)

**Table 5:** Response Patterns to Hyperbaric Oxygen Therapy in Patients with Different Brain Lesion Locations.

Lesion Location Grouping	Number of Cases	Pre-Treatment fNIRS Activation Features	Changes in fNIRS Activation Features Post-Treatment	Change in Number of Activated Channels Post-Treatment (Mean ± Standard Deviation)
Motor Cortex Pathway Group	15	Contralateral motor cortex activation is weak or absent.	Ipsilateral motor cortex and ipsilateral cerebellum exhibit compensatory activation.	+5.3 ± 2.1
Somatosensory Cortex Pathway Group	12	Contralateral motor cortex activation is diffuse, while somatosensory cortex activation is restricted.	Contralateral motor cortex activation is concentrated; compensatory activation occurs in the sensory association area.	+4.1 ± 1.8
Prefrontal-Subcortical Group	16	Insufficient prefrontal cortex activation and weakened connectivity with the motor cortex.	The activation range of the prefrontal cortex and its connectivity with the motor cortex are enhanced.	+6.7 ± 2.4

**Table 6:** Changes in Clinical Functional Scores of Patients Before and After Hyperbaric Oxygen Therapy (Mean ± Standard Deviation).

Clinical Assessment Tools	Pre-Treatment (n = 43)	Post-Treatment (n = 43)	Statistical Value	*p*-Value
Brunnstrom Staging (I–VI)	3.2 ± 0.9	3.8 ± 0.8	Z = −4.112	<0.001
Fugl-Meyer Assessment (Upper Limb)	28.5 ± 10.2	35.4 ± 9.6	t = −8.745	<0.001
ADL Score (Barthel Index)	45.6 ± 12.8	58.3 ± 11.5	t = −7.892	<0.001

## Data Availability

Due to ethical restrictions and the need to protect patient privacy, the data supporting the findings of this study are not publicly available.
